# Dupilumab induces a significant decrease of food specific immunoglobulin E levels in pediatric atopic dermatitis patients

**DOI:** 10.1002/clt2.12381

**Published:** 2024-07-17

**Authors:** Lisa P. van der Rijst, Michelle S. Hilbrands, Nicolaas P. A. Zuithoff, Marjolein S. de Bruin‐Weller, André C. Knulst, Thuy‐My Le, Marlies de Graaf

**Affiliations:** ^1^ Department of Dermatology and Allergology University Medical Center Utrecht Wilhelmina Children's Hospital Utrecht The Netherlands; ^2^ Department of Dermatology and Allergology National Expertise Center for Atopic Dermatitis University Medical Center Utrecht Utrecht The Netherlands; ^3^ Julius Center for Health Sciences and Primary Care University Medical Center Utrecht Utrecht The Netherlands

**Keywords:** atopic dermatitis, children, dupilumab, food allergy, pediatric, specific immunoglobulin E

To the Editor,

Atopic dermatitis (AD) and food allergy (FA) are common chronic diseases that have a major impact on quality of life and socio‐economic burden.^1^, ^2^ AD is strongly associated with the development of immunoglobulin E (IgE)‐mediated FA.^3^, ^4^, ^5^ The associated immune response involves allergen specific T helper type 2 cells inducing a pro‐inflammatory cytokine release, including interleukin (IL)‐4 and IL‐13, thereby causing initiation of B cell immunoglobulin class switching to specific IgE (sIgE).^4^ Dupilumab, a human monoclonal antibody that is approved for treatment of (moderate to) severe AD in children from the age of 6 months, blocks the IL‐4 and IL‐13 signaling pathway.^6^ Spekhorst et al. showed that dupilumab induces a profound decrease in sIgE levels of several food allergens in adult AD patients with comorbid FA, highlighting the positive effect of blocking IL‐4 and IL‐13 signaling on sIgE levels.^5^ As FA often develops at a young age, and the effect of dupilumab on food sIgE levels in pediatric patients remains unclear, it is of particular interest to evaluate the effect of dupilumab in this young patient population. Therefore, the aim of this study was to investigate the effect of dupilumab on food sIgE levels of 10 common allergens in food allergic pediatric patients with moderate to severe AD.

Pediatric AD patients (aged 4–17 years) treated with dupilumab with a suggestive clinical history of FA for peanut, hazelnut, cashew nut, pistachio, almond, walnut, hen's egg, cow's milk, kiwi, and/or apple with a corresponding positive sIgE (≥0.35 kU/L) at the start of treatment (baseline), were included. Patients who were never exposed to specific food allergens, due to severe IgE‐mediated reactions to other food allergens (e.g., hazelnut, leading to avoidance of other nuts) or to parental anxiety (e.g., due to severe parental FA), with a corresponding positive sIgE, were also included. sIgE levels were measured at baseline and at least once during 1 year of follow‐up. Data were extracted from the prospective BioDay registry between August 2019 and July 2023. A covariance pattern model was used to analyze the development of (s)IgE values over time. All analyses were performed for each food separately using a covariance pattern model (detailed explanation of methods is described in Supporting Information S1).

A total of 36 pediatric patients with a mean age of 12.5 (standard deviation ±3.6) years were included (Table 1). A total of 120 FAs, with 1008 corresponding sIgE samples, were identified (Table S1). Peanut (18.3%) and hazelnut (16.7%) were the most common foods to which patients were sensitized. Results of baseline food sIgE levels stratified by severity of FA are shown in Table S2. Two (5.6%) patients discontinued dupilumab treatment at a mean treatment duration of 16.5 weeks.

**TABLE 1:** Baseline characteristics.

Characteristic	
Total, *n*	36
Gender (F), *n* (%)	21 (58.3)
Age (years), mean (SD)	12.5 ± 3.6
BMI, mean (SD)	19.5 ± 4.2
Age AD onset (years), mean (SD)	0.8 ± 2.0
AD severity at inclusion	
EASI score, mean (SD)	21.8 ± 12.5
IGA score, mean (SD)	3.4 ± 0.7
Number of food allergies, *n* (%)	
1	11 (30.6)
≥2	25 (69.4)
Food allergies, total, *n*	120
Peanut, *n* (%)	22 (18.3)
Hazelnut, *n* (%)	20 (16.7)
Cashew nut, *n* (%)	14 (11.7)
Almond, *n* (%)	11 (9.2)
Walnut, *n* (%)	12 (10.0)
Pistachio, *n* (%)	9 (7.5)
Hen's egg, *n* (%)	9 (7.5)
Cow's milk, *n* (%)	7 (5.8)
Kiwi, *n* (%)	9 (7.5)
Apple, *n* (%)	7 (5.8)
Atopic comorbidity	
Allergic asthma, *n* (%)	21 (58.3)
Allergic rhinitis, *n* (%)	33 (91.7)
Allergic conjunctivitis, *n* (%)	19 (52.8)
Positive family history for atopic diseases, ≥1, *n* (%)	34 (94.4)
Atopic dermatitis, *n* (%)	30 (83.3)
Allergic asthma, *n* (%)	18 (50.0)
Allergic rhinitis, *n* (%)	25 (69.4)
Food allergy, *n* (%)	6 (16.7)
Total IgE (kU/L), median (IQR)	3786.0 (1344.0–5001.0)
Missing, *n* (%)	7 (19.4)
Serum TARC levels (pg/mL), median (IQR)	1950.0 (820.0–7260.0)
Missing, *n* (%)	1 (2.8)
Eosinophil levels (×10^9^/L), median (IQR)	0.6 (0.4–0.9)
Inhalant allergy screening, positive, *n* (%)	29 (80.6)
House dust mite (*n* = 26)	24 (92.3)
Birch pollen (*n* = 25)	24 (96.0)
Timothy grass pollen (*n* = 26)	25 (96.2)
Mugwort (*n* = 17)	13 (76.5)
Aspergillus fumigatus (*n* = 17)	14 (82.4)
Cat (*n* = 24)	21 (87.5)
Dog (*n* = 24)	24 (100.0)
Missing, *n* (%)	7 (19.4)

Abbreviations: AD, atopic dermatitis; BMI, body mass index; EASI, eczema area and severity index; IGA, Investigator's Global Assessment; IgE, immunoglobulin E; IQR, interquartile range; SD, standard deviation; TARC, thymus and activation regulated chemokine.

A significant percentage decrease was observed for all food allergens during 1 year of treatment, with the most profound decrease in the first 16 weeks of treatment (Figure 1). In all food allergens (sIgE extracts and components), a decrease ranging from 70.5% (95% CI: 37.1–86.1, for apple) to 82.5% (95% CI: 75.0–87.7, for cashew nut) was observed. Figure S1 shows the significant decrease in estimated median sIgE levels over time for all food allergens, ranging from 3.1 kU/L (95% CI: 2.2–4.2, for almond) to 33.7 kU/L (95% CI: 20.3–55.8, for hazelnut) at baseline and 0.8 kU/L (95% CI: 0.5–1.2, for almond) to 9.8 kU/L (95% CI: 6.0–15.9, for hazelnut) after 1 year of treatment. Higher baseline sIgE levels did not correspond to faster or slower decrease than lower baseline sIgE levels. In addition, subgroup analyses revealed no significant differences in the percentage decrease in sIgE levels between patients sensitized to one food and those sensitized to multiple foods (data not shown). However, these results were based on a small sample size.

**FIGURE 1:**
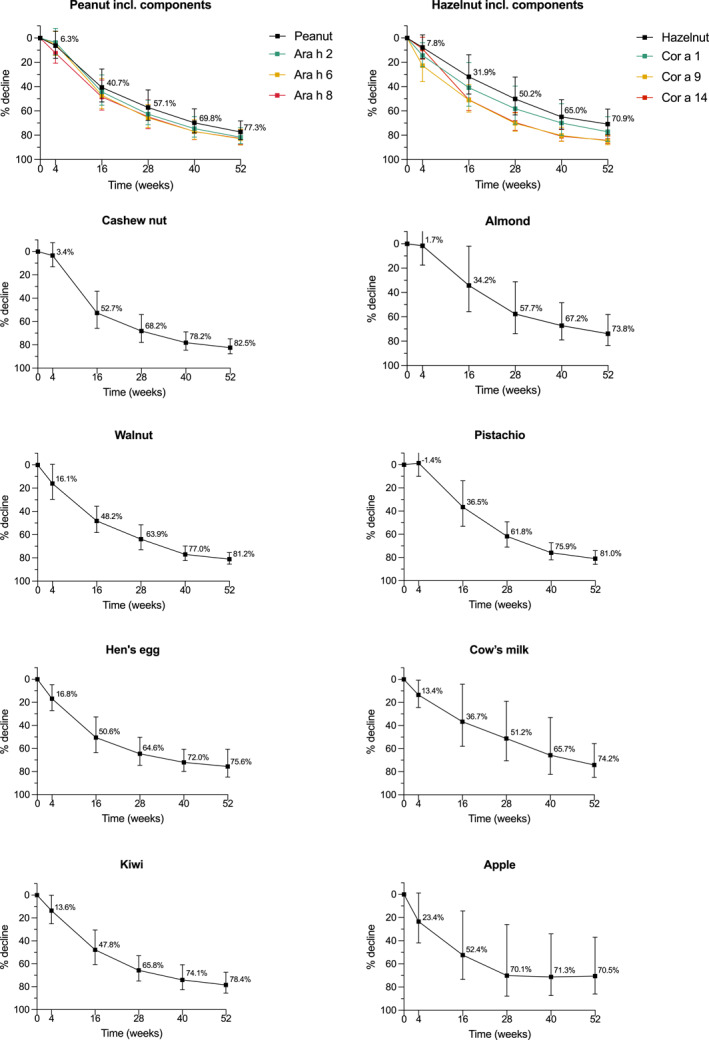
Estimated percentage decrease of median sIgE levels during 1 year of dupilumab treatment in pediatric patients with moderate to severe AD for peanut, hazelnut, cashew nut, almond, walnut, pistachio, hen's egg, cow's milk, kiwi, and apple. Error bars indicate the 95% confidence interval. AD, atopic dermatitis; sIgE, specific immunoglobulin E.

Total IgE levels decreased with 86.7% (95% CI: 70.2–94.1) after 1 year of treatment (Figure S2). Calculated ratios of sIgE to total IgE for various food allergens did not show significant changes over time, except for hen's egg (data not shown). However, ratios were based on limited statistical power and debated prognostic value.

This study shows a larger percentage decrease of sIgE levels compared to what was previously reported in food allergic adult AD patients treated with dupilumab.^5^ Spekhorst et al. reported an overall percentage decrease in food sIgE levels of 53.0%–62.9% after 1 year and 85.3%–86.9% after 3 years of dupilumab treatment.^5^ This difference may be age‐related and/or may be a result of the higher baseline food sIgE levels in children compared to adult AD patients.

Several studies indicate that decreasing sIgE‐levels could be a surrogate marker for the probability of developing clinical food tolerance.^7^, ^8^, ^9^ Nevertheless, it remains unclear whether the reduction of sIgE below a certain threshold leads to tolerance. Dupilumab, blocking the IL‐4/IL‐13 pathway, may play a key role in preventing IgE class switching, resulting in lower sIgE levels and potentially diminished severity of FA. Only one case report describes the development of food tolerance during dupilumab treatment for canned corn and pistachio in an adult AD patient, confirmed by an oral food challenge.^7^ Prospective studies including OFCs before, during and after treatment are needed to objectify whether dupilumab treatment leads to a higher threshold and/or less severe FA symptoms, the possible relation with sIgE levels, and whether this effect persists after treatment discontinuation. Furthermore, future studies will need to evaluate the effect of dupilumab treatment at a younger age (>6 months of age). Potentially, treatment at young age may influence the development of FA, by early improvement of epidermal barrier dysfunction and reduction of transepidermal water loss, and prevention of pro‐inflammatory cytokine release causing B cell immunoglobulin class switching to sIgE.^4^

In conclusion, this is the first study showing a profound decrease of sIgE levels in 10 common food allergens in food allergic pediatric patients with moderate to severe AD, ranging from 70.5% to 82.5% after 1 year of dupilumab treatment. These findings endorse an additional positive effect of dupilumab on multiple FAs in pediatric AD patients with comorbid FA. To elucidate the role of dupilumab in the treatment of FA, further understanding of the clinical and immunological effects of dupilumab in pediatric patients is needed.

## AUTHOR CONTRIBUTIONS

**Lisa P. van der Rijst**: Conceptualization; data curation; formal analysis; investigation; methodology; visualization; writing ‐ original draft; writing ‐ review & editing. **Michelle S. Hilbrands**: Data curation; formal analysis; investigation; methodology; writing ‐ original draft. **Nicolaas P. A. Zuithoff**: Methodology; writing ‐ review & editing; software; validation. **Marjolein S. de Bruin‐Weller**: Conceptualization; project administration; resources; supervision; writing ‐ review & editing; funding acquisition. **André C. Knulst**: Writing ‐ review & editing; supervision. **Thuy‐My Le**: Conceptualization; methodology; supervision; validation; visualization; writing ‐ review & editing; writing ‐ original draft. **Marlies de Graaf**: Conceptualization; data curation; investigation; methodology; resources; writing ‐ original draft; writing ‐ review & editing; supervision; visualization; funding acquisition.

## CONFLICT OF INTEREST STATEMENT

Lisa P. van der Rijst has been a speaker for AbbVie and Novartis; Michelle S. Hilbrands has nothing to disclose; Nicolaas P. A. Zuithoff has nothing to disclose; Marjolein S. de Bruin‐Weller has been a consultant, advisory board member, and/or speaker for AbbVie, Almirall, Amgen, Aslan, Eli Lilly, Galderma, Janssen, Leo Pharma, Pfizer, Regeneron Pharmaceuticals, and Sanofi; André C. Knulst has been a consultant, advisory board member, and/or speaker for ALK, Thermofisher, Nutricia/Danone, EUROIIMUN, DBV, and Novartis; Thuy‐My Le has been an advisory board member or speaker for Thermo Fisher Scientific, Novartis and Abbvie; Marlies de Graaf has been a consultant, advisor and/or speaker for AbbVie, Almirall, Eli Lilly, Janssen, Leo Pharma, Novartis, Pfizer, Regeneron Pharmaceuticals, and Sanofi.

## CONSENT FOR PUBLICATION

Not applicable.

## Supporting information

Supporting Information S1

## Data Availability

The data that support the findings of this study are available on request from the corresponding author, MdG. The data are not publicly available due to privacy restrictions.
