# Supplementary material for: Dupilumab induces a significant decrease of food specific immunoglobulin E levels in pediatric atopic dermatitis patients
Source: Clin Transl Allergy. 2024 Jul 17;14(7):e12381. doi: 10.1002/clt2.12381 (PMC11254451; doi:10.1002/clt2.12381)
Supplement: Supplementary file 1 — Supporting Information S1 [file CLT2-14-e12381-s001.docx]

#### Supporting Information

### **Detailed explanation of methods**

### Clinical data

Data on FA (type of food, patient-reported symptoms, severity of reaction) were collected by an experienced physician at the start of dupilumab treatment (baseline). The severity of FA was assessed and classified following the ordinal Food Allergy Severity Score (oFASS)-3; mild = oral allergy symptoms, moderate = skin reactions and gastrointestinal symptoms, severe = respiratory and cardiovascular symptoms, taking the most severe reaction per food into account.[^1^](#_ENREF_1)

### (s)IgE data collection

In patients allergic to peanut and/or hazelnut, components were measured: Ara h 2, Ara h 6, and Ara h 8 and/or Cor a 1, Cor a 9, and Cor a 14, respectively. Total IgE levels were measured at baseline and 16 and 52 weeks of treatment. For the calculation of data, sIgE levels of >100 kU/L were defined as 101 kU/L, and total IgE levels of >5000 kU/L were defined as 5001 kU/L.

### Statistical analysis

A covariance pattern model was used to analyze the development of (s)IgE levels over time for each allergen separately. This is a linear regression with a residual (i.e. GEE-type) covariance matrix to correct for multiple measurements of the outcome over time.[^2^](#_ENREF_2) Time (i.e. the number of weeks between baseline and sample collection) was included as a categorical determinant in the model, to avoid an assumption of a linearity. P-values for time were based on likelihood ratio tests. Initial analysis of the (s)IgE distribution and residual analysis indicated a deviation from normality assumption, therefore (s)IgE levels were log-transformed in subsequent analyses.

For ease of clinical interpretation, mean log-transformed (s)IgE values were derived from the results of the analyses, these values were transformed back to the original scale, thus providing estimated median (s)IgE values (with 95% CIs).[^3^](#_ENREF_3) Regression coefficients and 95% CIs were transformed to a percentage decline compared to baseline. P-values of <0.05 were regarded as statistically significant. Data were analyzed using IBM SPSS Statistics version 27.0.0.0 and SAS 9.4 (SAS Institute Inc., Cary, NC).

**References**

1. Fernández-Rivas M, Gómez García I, Gonzalo-Fernández A, Fuentes Ferrer M, Dölle-Bierke S, Marco-Martín G, et al. Development and validation of the food allergy severity score. Allergy. 2022;77(5):1545-58.

2. Garrett M. Fitzmaurice NML, James H. Ware. Applied Longitudinal Analysis. Wiley Series in Probability and Statistics. Hoboken, NJ: Wiley-Interscience 2004. p. i-xxv.

3. David J. Pasta MGC. Estimating Standard Errors for CLASS Variables in Generalized Linear Models Using PROC IML Statistics and Data Analysis.1-6.

**Table S1**. Number of total and positive food sIgE samples measured during dupilumab treatment.

|  | **Total** | | **Baseline** | | | **Week 4** | | **Week 16** | | | **Week 28** | | | **Week 40** | | | **Week 52** | |
| --- | --- | --- | --- | --- | --- | --- | --- | --- | --- | --- | --- | --- | --- | --- | --- | --- | --- | --- |
|  | Samples  (n) | Positive^‡^  (n, %) | Samples  (n) | Positive^‡^  (n, %) | Samples  (n) | | Positive^‡^  (n, %) | Samples  (n) | Positive^‡^  (n, %) | Samples  (n) | | Positive^‡^  (n, %) | Samples  (n) | | Positive^‡^  (n, %) | Samples  (n) | | Positive^‡^  (n, %) |
| Peanut | 94 | 93 (98.9) | 20 | 20 (100.0) | 14 | | 14 (100.0) | 19 | 19 (100.0) | 14 | | 14 (100.0) | 12 | | 12 (100.0) | 15 | | 14 (93.3) |
| Ara h 2 | 89 | 87 (97.8) | 20 | 20 (100.0) | 13 | | 13 (100.0) | 18 | 17 (94.4) | 13 | | 13 (100.0) | 11 | | 11 (100.0) | 14 | | 13 (92.9) |
| Ara h 6 | 88 | 82 (93.2) | 19 | 19 (100.0) | 13 | | 13 (100.0) | 18 | 16 (88.9) | 13 | | 13 (100.0) | 11 | | 10 (90.9) | 14 | | 11 (78.6) |
| Ara h 8 | 82 | 79 (96.3) | 17 | 17 (100.0) | 12 | | 12 (100.0) | 16 | 15 (93.8) | 13 | | 13 (100.0) | 10 | | 10 (100.0) | 14 | | 12 (85.7) |
| Hazelnut | 89 | 89 (100.0) | 19 | 19 (100.0) | 14 | | 14 (100.0) | 18 | 18 (100.0) | 14 | | 14 (100.0) | 11 | | 11 (100.0) | 13 | | 13 (100.0) |
| Cor a 1 | 82 | 81 (98.8) | 16 | 16 (100.0) | 14 | | 14 (100.0) | 15 | 15 (100.0) | 14 | | 14 (100.0) | 10 | | 10 (100.0) | 13 | | 12 (92.3) |
| Cor a 9 | 73 | 55 (75.3) | 15 | 15 (100.0) | 11 | | 8 (72.7) | 14 | 10 (71.4) | 12 | | 9 (75.0) | 10 | | 7 (70.0) | 11 | | 6 (54.5) |
| Cor a 14 | 61 | 50 (82.0) | 12 | 12 (100.0) | 9 | | 8 (88.9) | 12 | 10 (83.3) | 10 | | 7 (70.0) | 8 | | 6 (75.0) | 10 | | 7 (70.0) |
| Almond | 61 | 61 (100.0) | 12 | 12 (100.0) | 10 | | 10 (100.0) | 10 | 10 (100.0) | 10 | | 10 (100.0) | 10 | | 10 (100.0) | 9 | | 9 (100.0) |
| Cashew nut | 62 | 53 (85.5) | 14 | 14 (100.0) | 8 | | 8 (100.0) | 12 | 11 (91.7) | 9 | | 7 (77.8) | 10 | | 7 (70.0) | 9 | | 6 (66.7) |
| Pistache | 36 | 32 (88.9) | 8 | 8 (100.0) | 5 | | 5 (100.0) | 6 | 5 (83.3) | 5 | | 4 (80.0) | 6 | | 5 (83.3) | 6 | | 5 (83.3) |
| Walnut | 56 | 56 (100.0) | 12 | 12 (100.0) | 8 | | 8 (100.0) | 9 | 9 (100.0) | 9 | | 9 (100.0) | 9 | | 9 (100.0) | 9 | | 9 (100.0) |
| Kiwi | 40 | 40 (100.0) | 9 | 9 (100.0) | 8 | | 8 (100.0) | 8 | 8 (100.0) | 5 | | 5 (100.0) | 4 | | 4 (100.0) | 6 | | 6 (100.0) |
| Apple | 22 | 21 (95.5) | 6 | 6 (100.0) | 5 | | 5 (100.0) | 4 | 4 (100.0) | 4 | | 3 (75.0) | 2 | | 2 (100.0) | 1 | | 1 (100.0) |
| Cow’s milk | 35 | 34 (97.1) | 8 | 8 (100.0) | 7 | | 6 (85.7) | 7 | 7 (100.0) | 5 | | 5 (100.0) | 4 | | 4 (100.0) | 4 | | 4 (100.0) |
| Hen’s egg | 38 | 32 (84.2) | 8 | 8 (100.0) | 8 | | 7 (87.5) | 8 | 7 (87.5) | 5 | | 4 (80.0) | 4 | | 3 (75.0) | 5 | | 3 (60.0) |
| Total | 1008 | 945 (93.8) | 215 | 215 (100.0) | 159 | | 153 (96.2) | 194 | 181 (93.3) | 155 | | 144 (92.9) | 132 | | 121 (91.7) | 153 | | 131 (85.6) |

^‡^ Levels of ≥0.35 kU/L were considered as positive.

**Table S2.** Baseline median (IQR) specific IgE level (kU/L) per food and FA severity (oFASS-3 classification)

|  |  | Severity according to oFASS classification^‡^ | | | |
| --- | --- | --- | --- | --- | --- |
|  | **Total** | **Mild** | **Moderate** | **Severe** | **Unknown^¥^** |
| **Total**, n (%) | 120 (100.0) |  |  |  |  |
| **Peanut allergy**, n (%)  Extract  Ara h 2  Ara h 6  Ara h 8 | 22  35.9 (4.9-63.6)  25.5 (5.6-48.8)  17.9 (3.7-40.0)  27.8 (12.6-55.0) | 0 (0.0)  NA | 13 (59.1)  21.0 (3.5-61.0)  20.7 (5.6-54.5)  17.9 (1.2-40.0)  25.8 (13.3-53.0) | 8 (36.4)  40.4 (6.0-79.0)  26.9 (2.3-45.0)  12.3 (3.7-21.3)  38.0 (19.8-80.5) | 1 (4.5)  59.5 (59.5-59.5)  23.7 (23.7-23.7)  51.8 (51.8-51.8)  11.9 (11.9-11.9) |
| **Hazelnut**, n (%)  Extract  Cor a 1  Cor a 9  Cor a 14 | 20  47.0 (15.0-81.0)  58.0 (41.7-97.8)  4.0 (1.2-10.6)  18.2 (6.7-44.1) | 1 (5.0)  101.0 (101.0-101.0)  101.0 (101.0-101.0)  1.2 (1.2-1.2)  47.0 (47.0-47.0) | 11 (55.0)  39.0 (14.3-81.8)  56.0 (34.0-95.5)  10.6 (2.0-50.0)  17.1 (5.8-36.0) | 5 (25.0)  29.1 (3.9-70.0)  53.0 (38.0-NA)  2.7 (0.6-8.4)  5.5 (0.8-NA) | 3 (15.0)  61.6 (54.0-NA)  60.0 (43.9-NA)  5.9 (0.9-NA)  46.8 (19.3-NA) |
| **Cashew** nut, n (%)  Extract | 14  10.1 (1.3-62.7) | 1 (7.1)  71.0 (71.0-71.0) | 5 (35.7)  1.1 (1.0-56.0) | 5 (35.7)  9.9 (2.6-10.4) | 3 (21.4)  60.6 (6.9-NA) |
| **Almond**, n (%)  Extract | 11  3.4 (1.7-5.3) | 0 (0.0)  NA | 6 (54.5)  3.4 (1.8-4.7) | 3 (27.3)  1.7 (1.4-NA) | 2 (18.2)  4.3 (2.2-NA) |
| **Walnut**, n (%)  Extract | 12  20.1 (7.5-55.8) | 0 (0.0)  NA | 4 (33.3)  28.3 (4.4-55.8) | 5 (41.7)  14.4 (7.9-50.9) | 3 (25.0)  39.2 (2.1-NA) |
| **Pistachio**, n (%)  Extract | 9  14.0 (6.4-43.8) | 0 (0.0)  NA | 3 (33.3)  7.6 (0.6-NA) | 5 (55.6)  14.0 (7.2-59.9) | 1 (11.1)  35.6 (35.6-35.6) |
| **Hen’s egg**, n (%)  Extract | 9  17.4 (5.5-53.9) | 1 (11.1)  0.9 (0.9-0.9) | 7 (77.8)  17.4 (10.2-25.8) | 1 (11.1)  101.0 (101.0-101.0) | 0 (0.0)  NA |
| **Cow’s milk**, n (%)  Extract | 7  72.0 (6.5-88.0) | 0 (0.0)  NA | 4 (57.1)  44.1 (6.8-85.5) | 3 (42.9)  72.0 (6.5-NA) | 0 (0.0)  NA |
| **Kiwi**, n (%)  Extract | 9  16.0 (3.9-21.0) | 4 (44.4)  3.9 (1.5-7.5) | 5 (55.6)  20.7 (17.3-22.1) | 0 (0.0)  NA | 0 (0.0)  NA |
| **Apple**, n (%)  Extract | 7  6.4 (4.2-9.6) | 5 (71.4)  8.8 (2.6-10.9) | 2 (28.6)  5.7 (5.0-NA) | 0 (0.0)  NA | 0 (0.0)  NA |

^‡^ Mild = oral allergy symptoms; Moderate = skin reactions and gastrointestinal symptoms; Severe = respiratory and cardiovascular symptoms.

*Abbreviations:* IQR = Interquartile range, NA = not applicable. ¥ Unknown due to non-introduced foods. Missing baseline specific IgE: peanut extract = 2, Ara h 2 = 1, Ara h 6 = 2, Ara h 8 = 2, Hazelnut (incl. components) = 1, Almond = 1, Walnut = 1.


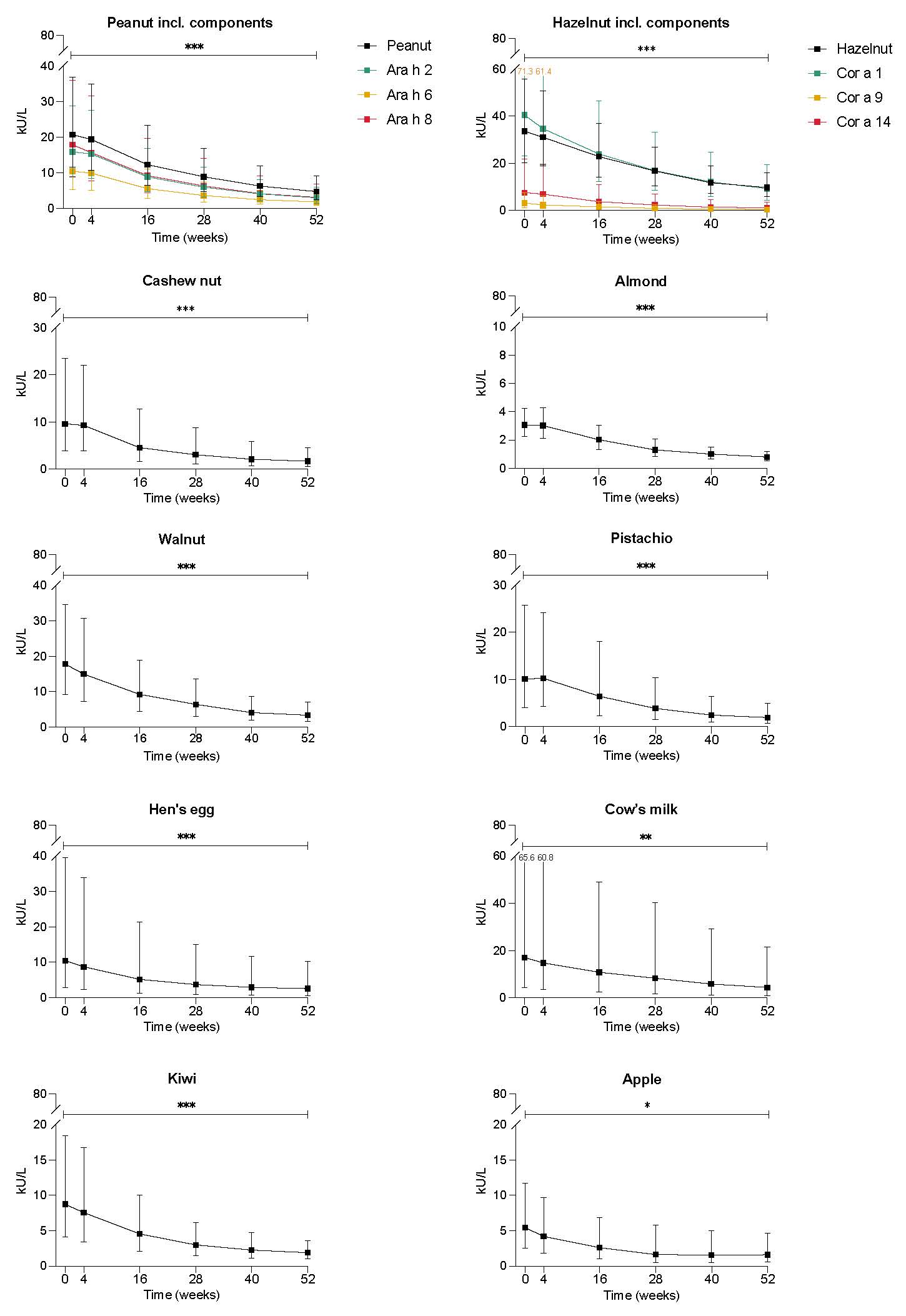
**Figure S1**. Estimated median sIgE levels during 1 year of dupilumab treatment in patients with moderate to severe AD for peanut, hazelnut, cashew nut, almond, walnut, pistachio, hen’s egg, cow’s milk, kiwi, and apple.

Error bars indicate the 95% confidence interval. 🞸 *P*<.05, 🞸🞸 *P*<.01, 🞸🞸🞸 *P*<.001.

**Figure S2.** Estimated percentage decrease and absolute median total IgE levels during 1 year of dupilumab treatment in pediatric patients with moderate to severe AD.


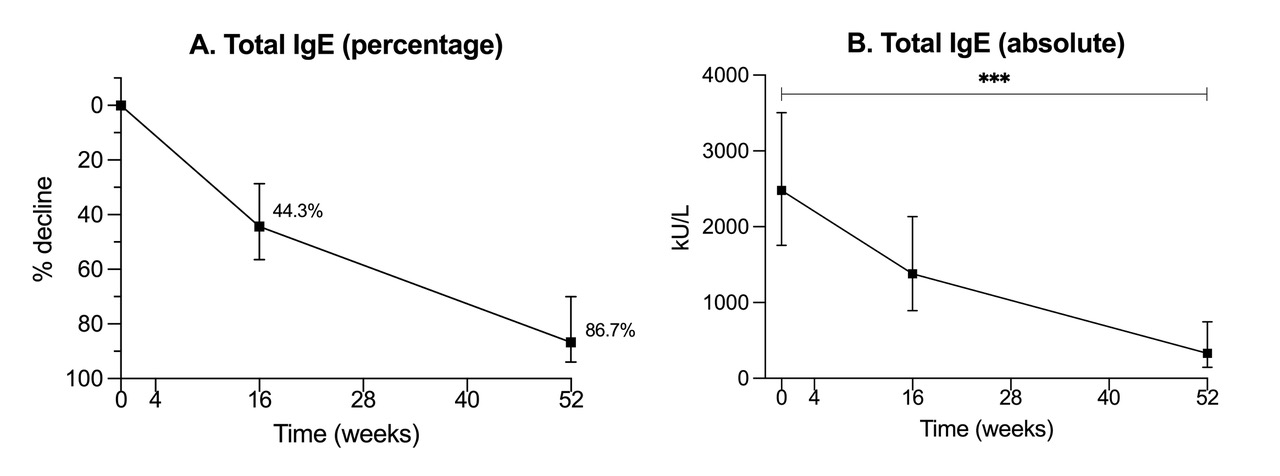


Error bars indicate the 95% confidence interval. 🞸🞸🞸 *P*<.001.
